# Importance of *Candida* infection and fluconazole resistance in women with vaginal discharge syndrome in Namibia

**DOI:** 10.1186/s13756-022-01143-6

**Published:** 2022-08-15

**Authors:** Cara M. Dunaiski, Marleen M. Kock, Hyunsul Jung, Remco P. H. Peters

**Affiliations:** 1 Department of Health and Applied Sciences, Namibia University of Sciences and Technology, Windhoek, Namibia; 2 Department of Medical Microbiology, University of Pretoria, Prinshof Campus, Pathology Building, Room 3-11, Pretoria, South Africa; 3 Tshwane Academic Division, National Health Laboratory Service, Pretoria, South Africa; 4 Division of Medical Microbiology, University of Cape Town, Cape Town, South Africa; 5 Research Unit, Foundation for Professional Development, East London, South Africa

**Keywords:** *Candida albicans*, Non-albicans *Candida* species, Vaginal discharge syndrome, Antifungal susceptibility testing, Sexually transmitted infections, Namibia, Sub-Saharan Africa

## Abstract

**Background:**

Vaginal discharge syndrome (VDS) is a common condition. Clinical management targets sexually transmitted infections (STIs) and bacterial vaginosis (BV); there is limited focus on *Candida* infection as cause of VDS. Lack of *Candida* treatment coverage and, if present, antifungal resistance may result in VDS treatment failure. This study aimed to determine the prevalence of *Candida* infection, antifungal resistance, and coinfections in Namibian women with VDS.

**Methods:**

A cross-sectional study was performed using 253 vaginal swabs from women with VDS in Namibia. Demographic data was collected, and phenotypic and molecular detection of *Candida* species was performed followed by fluconazole susceptibility testing of *Candida* isolates. BV was diagnosed using Nugent score microscopy; molecular detection of *Chlamydia trachomatis*, *Neisseria gonorrhoeae* and *Trichomonas vaginalis* was performed.

**Results:**

*Candida* species was detected in 110/253 women (43%). Ninety women (36%) had *Candida albicans* and 24 (9.5%) had non-albicans *Candida* species. The non-albicans species detected were 19 (17%) *Candida glabrata*, 4.0 (3.5%) *Candida krusei*, and 1.0 (0.9%) *Candida parapsilosis*. *Candida albicans* were more frequently isolated in younger (*p* = 0.004) and pregnant women (*p* = 0.04) compared to non-albicans *Candida* species. Almost all (98%) *Candida albicans* isolates were susceptible to fluconazole while all non-albicans *Candida* species were fluconazole resistant. STIs were diagnosed in 92 women (36%): 30 (12%) with *C. trachomatis*, 11 (4.3%) *N. gonorrhoeae*, and 70 (28%) *T. vaginalis*; 98 (39%) women had BV. *Candida* infection alone was diagnosed in 30 women (12%), combined with STIs in 42 women (17%) and was concurrent with BV in 38 women (15%). *Candida* infection was more often detected in swabs from women without *C. trachomatis* detected (6.4% vs. 16%; OR 0.30; 95% CI 0.10–0.77, *p* = 0.006).

**Conclusions:**

The high prevalence of *Candida* infection, especially those due to non-albicans *Candida* species that are resistant to fluconazole, is a great concern in our setting and may lead to poor treatment outcomes. Access to microbiological testing for *Candida* species in the context of syndromic management is warranted.

## Background

Vaginal discharge syndrome (VDS) is the most common gynaecological condition among women of reproductive age [1]. Vulvovaginal candidiasis (VVC) is the most common aetiology of VDS, accounting for about 90% of symptomatic vaginal infections [2, 3]. Up to 75% of healthy women face symptomatic VVC at least once in their childbearing years, with some experiencing intermittent and often obdurate forms of the disease [4, 5]. A previous study from Namibia reported that symptomatic VVC was present in 61/335 (18.2%) of women newly diagnosed with human immunodeficiency virus (HIV) that were enrolled for antiretroviral therapy [6]. Additionally, recurrent VVC (RVVC) may occur in 10%-20% of women with symptomatic VVC [7]. In two recent studies, RVVC, defined as four or more symptomatic episodes per year, was estimated to occur in 37,390 females per year in Namibia and in over a million females per year in South Africa [8, 9]. There are several factors contributing to RVVC, which include treatment failure, co-infections and antifungal resistance [10].

The aetiology in more than 90% of the VVC cases is *Candida albicans* [11]. Non-albicans *Candida* species have emerged as an important aetiology of VVC as its prevalence and antifungal resistance is a mounting problem globally [10, 11]. The most significant of these non-albicans *Candida* species is *Candida glabrata* owing to its intrinsic resistance or low susceptibility to azoles [12]. Other than *C. glabrata*, *C. krusei, C. tropicalis, C. parapsilosis* and, very rarely, *Saccharomyces cerevisiae*, are other potential pathogens that may lead to VVC [13].

In addition to VVC, VDS may also be caused by sexually transmitted infections (STIs), such as *Chlamydia trachomatis, Trichomonas vaginalis, Neisseria gonorrhoeae*, and bacterial vaginosis (BV) [2, 3]. In Namibia, like other low- to middle-income countries (LMICs), syndromic management of VDS is the standard of care, where clinicians treat patients empirically, without aetiological diagnosis, based on a set of symptoms [14]. This approach is associated with under-treatment as STIs frequently remain asymptomatic, but over-treatment with unnecessary use of antibiotics also occurs [15, 16]. Determination of the microbiological aetiology of VDS is essential to guide empirical treatment algorithms and to guide effective prescription of antifungal drugs for presumed candidiasis. Most of such studies focus on STIs and do not include *Candida* species, or antifungal resistance [15, 17, 18]. Comprehensive evaluation of microbial aetiology of VDS is essential to better understand the occurrence of *Candida* species, as well as the intersection with STIs and BV. The aim of this study was to determine the prevalence of *Candida* species, fluconazole resistance and co-infections in swabs collected from women with VDS in Namibia.

## Methods

### Study design, setting and population

This cross-sectional study was conducted using 253 vaginal swabs collected from women with VDS between February and July 2021 at primary healthcare facilities across Namibia. The vaginal swabs were collected by healthcare workers, which were sent to the diagnostic laboratory for routine diagnostic testing.

The laboratory requisition forms were assessed for inclusion and exclusion in the study. Vaginal swabs from women aged 18 to 49 years of age with ‘vaginal discharge’ as diagnostic indication recorded by the clinician were included, while swabs were excluded if more than two weeks old.

### Detection and identification of Candida isolates

Vaginal swabs were inoculated on the chromogenic *Candida* agar plates (CHROMagar™, France), which were incubated (Thermo Scientific, USA) at 37 °C for 24–36 h. Colonies isolated on chromogenic *Candida* agar were used to identify *Candida* spp. according to colony colour, as per manufacturer’s instructions. In each test, the reference strain *C. albicans* American Type Culture Collection (ATCC) 14053 was used for quality control. If there were no visible colonies within 3 days, the sample was considered negative for *Candida*. Colonies were inoculated on the Sabouraud Dextrose Agar (SDA) plates (Oxoid, United Kingdom) in order to be stored with 50% glycerol (Merck, Germany) at − 20 °C prior to DNA extraction.

### Molecular confirmation of Candida species

Deoxyribonucleic acid (DNA) extraction and purification from vaginal swabs were performed using the Quick-DNA™ Fungal/ Bacterial Miniprep Kit (Zymo Research, USA) according to the manufacturer’s guidelines. Multiplex PCR was performed using the One Taq® Quick-Load 2 × Master Mix (New England BioLabs, USA), a universal *Candida* primer pair targeting *ITS1* and *ITS2* and *C. albicans* specific primers according to Rad et al*.* [19]. The PCR products were then analysed with the 50 bp DNA ladder (New England BioLabs, USA) by gel electrophoresis through a 2% agarose gel and ultraviolet visualisation [19]. *Candida albicans* ATCC 14053, *C. glabrata* CBS2175, *C. parapsilosis* CBS2195, *C. tropicalis* CBS94, and *C. krusei* CBS473 were included in each PCR reaction as positive controls; nuclease-free water (BioConcept, Switzerland) was used as negative control.

### Fluconazole antifungal susceptibility patterns of Candida isolates

The fluconazole susceptibility of *Candida* isolates was determined using the European Committee on Antimicrobial Susceptibility Testing (EUCAST) microbroth dilution (MBD) method to determine the minimum inhibitory concentration (MIC) [20]. The EUCAST breakpoints were used to assign the *Candida* species into the clinical categories “susceptible”, “intermediate” and “resistant” [20]. Quality control isolates included *C. parapsilosis* ATCC 22019 (susceptible) and *C. krusei* ATCC 6258 (resistant) [21].

### Detection of bacterial vaginosis

A Gram-stained vaginal smear prepared from vaginal swab was examined under a microscope and evaluated for BV by Nugent scoring [22, 23]. Nugent scores from 0 to 3 are considered as “Normal”; 4 to 6 as “Intermediate”; and 7 to 10 as “BV” [24].

### Detection of sexually transmitted infections

Molecular detection of *Chlamydia trachomatis* and *N. gonorrhoeae* was performed using the LightMix 480 HT CT/NG assay (TIB MOLBIOL, Berlin, Germany), while a validated in-house real time-PCR assay, as described elsewhere, was performed for detection of *T. vaginalis* [25].

### Statistical analyses

Data were captured into a study-specific Epi Info™ database version 7.2.4.0 (Centres for Disease Control and Prevention (CDC), USA) and exported into RStudio version 2021.09.1 (RStudio, USA) for analysis. Data are presented as absolute value with proportion, and median with range. The chi-squared test, with Fisher’s Exact if appropriate, was used to compare dichotomous variables between groups, while the Mann–Whitney test was used for continuous variables between groups. Logistic regression was used to calculate associations of age and pregnancy between *Candida albicans* and non-albicans *Candida* species isolated from women with yeast infections. A *p* value < 0.05 was considered statistically significant.

## Results

### Study population

A total of 253 vaginal swabs from women with VDS were included. The median age of these women was 29 years (interquartile range (IQR) 24–34), 58 (23%) were HIV-infected and 60 (24%) were pregnant.

*Candida* isolates were detected in vaginal swabs from 110 (43%; 110/253) women; there was no association with any of the demographic variables (Table 1). In addition, *Chlamydia trachomatis* was detected from 30 women (12%), *N. gonorrhoeae* from 11 (4.3%) and *T. vaginalis* from 70 (28%). Bacterial vaginosis was present in 98 women (39%) while 69 (27%) and 98 (39%) belonged to an intermediate and normal Nugent score category.

**Table 1:** Demographic factors and coinfections in women with and without *Candida* infection in Namibia

Characteristics	Total (*n* = 253)	*Candida* species isolated		COR	95% CI	*p* value
		Yes (*n* = 110)	No (*n* = 143)			
Median age in years (IQR)	29 (24–34)	28 (23–32)	30 (24–36)			0.11
*Pregnancy status*						
Pregnant	60 (24)	31 (28)	29 (20)	1.54	0.83–2.9	0.18
Not pregnant	193 (76)	89 (78)	114 (80)			
*HIV status*						
HIV-positive	58 (23)	23 (21)	35 (24)	0.82	0.43–1.5	0.55
HIV-negative	195 (77)	87 (79)	108 (76)			
*Chlamydia trachomatis*	30 (12)	7.0 (6.4)	23 (16)	0.30	0.10–0.77	0.006
*Neisseria gonorrhoea*	11 (4.0)	5.0 (4.5)	6.0 (4.2)	1.1	0.26–4.4	1.0
*Trichomonas vaginalis*	70 (28)	30 (27)	40 (28)	0.97	0.53–1.7	1.0
Bacterial vaginosis	98 (39)	38 (34)	60 (42)	0.73	0.42–1.3	0.24

Data are presented as number (n) with proportion (%) unless indicated otherwise

COR, crude odds ratio; CI, confidence interval; IQR, interquartile range; HIV, human immunodeficiency virus

Any *Candida* infection was less likely detected in swabs from women with *Chlamydia trachomatis* (6.4% vs. 16%; OR 0.30; 95% CI 0.10–0.77, *p* = 0.006). There was no relationship between *Candida* infection and age, HIV, *N. gonorrhoeae*, *T. vaginalis*, and BV.

### Distribution of Candida species recovered from vaginal swabs

Among the 110 women who tested positive for *Candida*, 114 *Candida* isolates were detected using culture methods, i.e. both *C. albicans* and *C. glabrata* were detected in four vaginal swabs. *C. albicans* was the most common isolate (*n* = 90, 79%), followed by the following non-albicans *Candida* species: *C. glabrata* (*n* = 19, 17%), *C. krusei* (*n* = 4, 3.5%) and *C. parapsilosis* (*n* = 1, 0.9%) (Table 2). Molecular methods confirmed phenotypic identification in all isolates (100% concordance).

**Table 2:** Distribution of 114 *Candida* species isolated from 110 swabs from women with vaginal discharge syndrome in Namibia

*Candida* species	No. of isolates	Prevalence (%)	95% CI
*C. albicans*	90	79	71–86
*C. glabrata*	19	17	11–25
*C. krusei*	4	3.5	1.4–8.7
*C. parapsilosis*	1	0.9	0.04–4.8
Total	114	100	

Data are presented as number (n) with proportion (%) unless indicated otherwise

CI, confidence interval

There were significant differences between women with *C. albicans* and non-albicans *Candida* spp. with regards to age (*p* = 0.01) and pregnancy (*p* = 0.002) (Table 3). Similar results were observed with the multivariate analysis, where both age (*p* = 0.004) and pregnancy (*p* = 0.04) were found to be independently associated with *C. albicans* isolated from vaginal swabs. *Candida albicans* are more likely to occur in pregnant women (adjusted odds ratio 1.9; 95% CI 1.0–3.5, *p* = 0.002) and in younger women, as *C. albicans* is less likely to occur with increasing age (adjusted odds ratio 0.94; 95% CI 0.90–0.98, *p* = 0.01).

**Table 3:** Demographic factors and coinfections in women with *Candida albicans* versus non-albicans species isolated from vaginal swabs in Namibia (n = 106)

Syndrome/infection	*Candida albicans* (n = 86)	Non-albicans *Candida* (n = 20)	COR	95% CI	*p* value
Median age in years (IQR)^a^	27.5 (23–32)	29.5 (23–37)	0.94	0.90–0.98	0.01
*Pregnancy*^*a*^					
Pregnant	27 (31)	0 (0)	1.9	1.0–3.5	0.002
Not pregnant	59 (690	20 (100)			
*HIV status*					
HIV-positive	17 (20)	2 (10)	2.2	0.45–21	0.52
HIV-negative	69 (80)	18 (90)			
*Chlamydia trachomatis*	7 (8.1)	0 (0)	–	–	0.34
*Neisseria gonorrhoea*	4 (4.7)	0 (0)	–	–	1.00
*Trichomonas vaginalis*	21 (24)	2 (10)	2.9	0.61–28	0.23
Bacterial vaginosis	32 (37)	5 (25)	1.8	0.54–6.8	0.44

Data are presented as number (n) with proportion (%) unless indicated otherwise

Four women with concurrent *Candida albicans* and non-albicans species are not included in this analysis

COR, crude odds ratio; CI, confidence interval; IQR, interquartile range; HIV, human immunodeficiency virus

^a^Multivariate analysis: adjusted odds ratio for age is 0.94 (95%CI 0.90–0.98, *p* = 0.01) and for pregnancy 1.9 (95% CI 1.0–3.5, *p* = 0.002)

### Fluconazole susceptibility pattern

The overall drug susceptibility pattern of *Candida* isolates against fluconazole is shown in Table 4. Fluconazole resistance was low in *C. albicans* isolates, but high in all non-albicans *Candida* isolates: *C. glabrata* (74%), *C. krusei* (100%) and *C. parapsilosis* (100%). There was no significant association between fluconazole susceptibility of *C. albicans* with demographic factors or coinfections.

**Table 4:** In vitro fluconazole susceptibility of *Candida* isolates (n = 114) collected from women with vaginal discharge in Namibia

*Candida* species	No. of isolates (%)	MIC range (mg/L)	MIC breakpoints^a^		
			Susceptible	Intermediate	Resistant
*C. albicans*	90 (79)	2–4	88 (98)	0 (0)	2 (2.0)
*C. glabrata*	19 (16)	0.001–16	0	5 (26)	14 (74)
*C. krusei*	4 (3.5)	–^b^	0	0	4 (100)
*C. parapsilosis*	1 (0.9)	> 4	0	0	1 (100)

^a^EUCAST (version 7.3.2) breakpoints were used for interpretation

^b^No breakpoints: *C. krusei* intrinsically resistant to azole antifungals

### Relationship between Candida species, STIs and BV

**Fig. 1:**
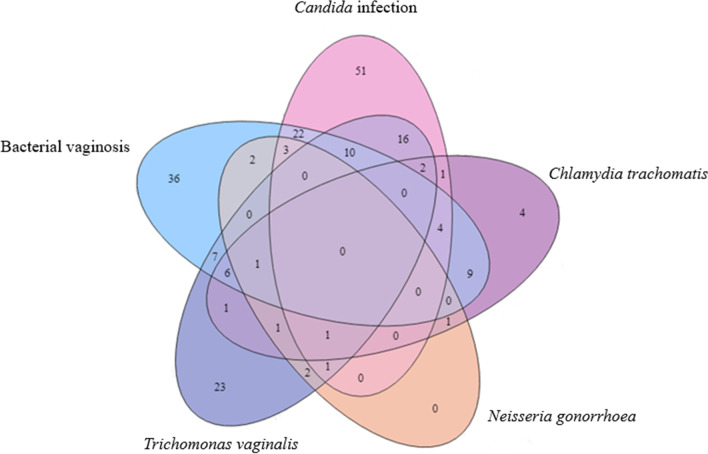
Overlapping microbial aetiology of vaginal discharge syndrome in women in Namibia

Multiple infections were common: two or more concurrent infections were detected in 86 (34%) women (Fig. 1). Vaginal infections caused by *Candida* alone occurred in 30/110 (27%) women. Concurrent infections occurred in 80//110 (73%) of women with *Candida* and included those with STIs (*n* = 42, 38%) including *T. vaginalis* (*n* = 30, 27%), *Chlamydia trachomatis* (*n* = 7, 6.4%) and *N. gonorrhoeae* (*n* = 5, 4.5%). In addition, BV co-occurred with *Candida* in 15% of the study population. Concurrent infections with BV (*n* = 86/98, 88%) included *T. vaginalis* (*n* = 24, 24%), *Chlamydia trachomatis* (*n* = 19, 19%) and *N. gonorrhoeae* (*n* = 5, 5.0%). However, neither *Candida* spp., *C. trachomatis*, *N. gonorrhoeae* nor *T. vaginalis* was detected in 52 women (21%).

## Discussion

Vaginal discharge syndrome (VDS) is a common cause of gynaecological visits among women in sub-Saharan Africa [1, 26, 27]. Most studies report only on specific microbiological aetiology of this condition, usually STIs, but only few have studied microbial aetiology comprehensively. Consequently, VVC and antifungal resistance is often not reported [1, 15, 17, 28]. This study provides a comprehensive analysis of microbial aetiology of VDS in Namibian women; it highlights a high prevalence of *Candida* species, including fluconazole-resistant non-albicans *Candida* species, and concurrence with STIs and BV.

*Candida* species were detected in 110 (43%) of swabs collected from women with VDS in our study making it the most common microbial aetiology. Studies from sub-Saharan Africa reported a wide range of *Candida* prevalence in women with VDS: 21% in Rwanda [29], 25% in Ethiopia [30], 26% in Mauritania [31], 29% in Senegal and Gabon [15, 32], 38% in Cameroon [33], 39% in Benin [34], 45% in South Africa [35], 49% Burkina Faso [36], 55% in Nigeria [37] and 66% in Tanzania [38]. The geographic differences in the reported prevalence described in different settings might be owing to environmental, behavioural, socioeconomic factors, as well differences in study methodologies [30].

In this cross-sectional study, no significant association with older age, pregnancy or HIV seropositive status was observed although these are known risk factors for VVC [39–43]. However, we did observe a significant association between the presence of *Candida* infection and detection of *Chlamydia trachomatis,* but not with the other STIs, where *C. trachomatis* was less likely to be detected in women with *Candida* (COR, 0.30, p = 0.006). A study by Kruppa and colleagues demonstrated a novel interaction between *Chlamydia trachomatis* and *C. albicans* via the binding of elementary bodies of *Chlamydia trachomatis* to *C. albicans* yeast and hyphal forms. This binding was shown to considerably decrease the capacity of *Chlamydia trachomatis* to infect human cervical epithelial cells, thereby decreasing its disease progression [44]. In contrast, another study illustrated that biofilms related to VVC may act as a reservoir for *Chlamydia trachomatis* [3]. STIs have been suggested in other studies as risk factor for VVC; however, this relationship should be further confirmed [45, 46].

In our study, like most other studies looking at the species distribution of *Candida*, the most prevalent *Candida* species isolated was *C. albicans*, followed by *C. glabrata* [47, 48]. *Candida glabrata* is the most relevant non-albicans *Candida* species, owing to its ability to develop acquired resistance subsequent to exposure to azole antifungals [11, 12, 49]. In our study, low rate of fluconazole resistance was found in *C. albicans* isolates (< 5%), but most non-albicans *Candida* isolates (*n* = 19, 17%) were fluconazole resistant. These findings are similar to reports from Africa and other parts of the world [33, 48]. Antifungal resistance of *Candida* spp. is a mounting problem universally [49–51]. High rates of fluconazole resistance has been demonstrated in several countries including China [52], Iran [53], Ethiopia [39], Peshawar [54], Brazil [55], Cameroon [33] and Uganda [48], to mention but a few. The use of azole antifungals may stimulate the selection of resistant subpopulations of *Candida* by shifting colonisation to more intrinsically resistant species, especially *C. krusei* or *C. glabrata* [56]. In our study, non-albicans species that were not susceptible to fluconazole were detected in 24/253 (9.5%) women with VDS, which is 22% of all women with *Candida* infection, highlighting the challenge in management of *Candida* species in the syndromic management context. Since pregnancy predisposes women to VVC, which in turn could increase the risk for poor pregnancy outcomes, it is reassuring that the pregnant women in this study all had *C. albicans* infection, and not fluconazole-resistant non-albicans *Candida*, and could therefore be adequately treated [57]. We observed an association between older age and the isolation of non-albicans species vs. *C. albicans* [4]. Similarly, some studies show that non-albicans species, such *C. glabrata*, are associated with older age when compared to *C. albicans*, which may be due to the exposure of several risk factors such as the use of hormonal contraceptives and broad spectrum antifungals [58, 59].

Our study demonstrates the complex microbial aetiology of VDS. Several women in this study experienced more than one infection, and some up to three infections at once, which may lead to overlapping diagnosis and conditions. Hence, these findings questions whether empirical approach for the management of VDS based on symptoms is appropriate or not [2].

This study has several limitations. First, limited demographic and clinical information was available from study participants due to collecting specimens submitted to the laboratory. Reliance on the information provided by the requesting clinician might have resulted in some misclassification. Second, the culture and molecular methods used target *Candida* species that cause vaginal infections; other *Candida* species might have been missed by these assays.

## Conclusion

This study highlights a high prevalence of VVC in women with VDS in Namibia. The high frequency of non-albicans *Candida* species that are resistant to fluconazole is a great concern and may contribute to poor treatment outcomes. Access to microbiological testing for *Candida* species in the context of syndromic management is warranted.

## Data Availability

The datasets used and/or analysed during the current study are available from the corresponding author on reasonable request.

## Abbreviations

AOR: Adjusted odds ratio
AMR: Antimicrobial resistance
AST: Antimicrobial susceptibility testing
ATCC: American Type Culture Collection
BV: Bacterial vaginosis
CDC: Centres of Disease Control and Prevention
CI: Confidence interval
COR: Crude odds ratio
DNA: Deoxyribonucleic acid
EUCAST: European Committee on Antimicrobial Susceptibility Testing
HIV: Human immunodeficiency virus
IQR: Interquartile range
LMICs: Low- to middle-income countries
MIC: Minimum inhibitory concentration
PCR: Polymerase chain reaction
RVVC: Recurrent vulvovaginal candidiasis
SDA: Sabouraud dextrose agar
STI: Sexually transmitted infection
VDS: Vaginal discharge syndrome
VVC: Vulvovaginal candidiasis
WHO: World Health Organization
